# Bridging the Gap between Psychophysiological and Audiological Factors in the Assessment of Tinnitus: An EEG Investigation in the Beta Band

**DOI:** 10.3390/brainsci14060570

**Published:** 2024-06-03

**Authors:** Bianca Maria Serena Inguscio, Dario Rossi, Giovanna Giliberto, Alessia Vozzi, Gianluca Borghini, Fabio Babiloni, Antonio Greco, Giuseppe Attanasio, Giulia Cartocci

**Affiliations:** 1 Department of Molecular Medicine, Sapienza University of Rome, 00161 Rome, Italy; biancams.inguscio@uniroma1.it (B.M.S.I.); dario.rossi@uniroma1.it (D.R.); giliberto.1795255@studenti.uniroma1.it (G.G.); gianluca.borghini@uniroma1.it (G.B.); fabio.babiloni@uniroma1.it (F.B.); 2 BrainSigns Srl, 00198 Rome, Italy; alessia.vozzi@brainsigns.com; 3 Department of Computer Science, Hangzhou Dianzi University, Hangzhou 310018, China; 4 Department of Sense Organs, Sapienza University of Rome, 00161 Rome, Italy; antonio.greco@uniroma1.it; 5 Head and Neck Department, Policlinico Umberto I, 00161 Rome, Italy; giuseppe.attanasio@uniroma1.it

**Keywords:** tinnitus, beta, electroencephalography (EEG), trait anxiety, state anxiety, hyperacusis, ecological listening task, networks, audiobook

## Abstract

Background: Despite substantial progress in investigating its psychophysical complexity, tinnitus remains a scientific and clinical enigma. The present study, through an ecological and multidisciplinary approach, aims to identify associations between electroencephalographic (EEG) and psycho-audiological variables. Methods: EEG beta activity, often related to stress and anxiety, was acquired from 12 tinnitus patients (TIN group) and 7 controls (CONT group) during an audio cognitive task and at rest. We also investigated psychological (SCL-90-R; STAI-Y; BFI-10) and audiological (THI; TQ12-I; Hyperacusis) variables using non-parametric statistics to assess differences and relationships between and within groups. Results: In the TIN group, frontal beta activity positively correlated with hyperacusis, parietal activity, and trait anxiety; the latter is also associated with depression in CONT. Significant differences in paranoid ideation and openness were found between groups. Conclusions: The connection between anxiety trait, beta activity in the fronto-parietal cortices and hyperacusis provides insights into brain functioning in tinnitus patients, offering quantitative descriptions for clinicians and new multidisciplinary treatment hypotheses.

## 1. Introduction

Perception of sounds and noises through the auditory system serves to help individuals learn about the external world, orient themselves, and maintain contact with it. However, some people may perceive sounds without an external source, a phenomenon known as tinnitus, from the Latin ‘*tinnire*’ (to ring). Tinnitus is the perception of sound within the human ear in the absence of external acoustic stimuli. This ‘phantom sound’ potentially causes disabling problems [1]. Individuals experiencing tinnitus report an unspecified acoustic sound like ringing but also buzzing, clicking, pulsations, and other noises [2]. The tinnitus-associated sufferance is defined as ‘tinnitus disorder’ and consists of emotional distress, cognitive dysfunction, and/or autonomic arousal (i.e., stress), leading to functional disability [3]. This phantom sound is often associated with comorbidities, especially in the auditory domain, such as hearing loss (HL) [4] and hyperacusis (increased sensitivity to perceived sound) [5], and studies suggest that the ability to perceive speech in noise is affected in individuals with tinnitus compared to those without tinnitus [6,7,8]. However, some tinnitus patients show normal hearing in conventional audiometry and do not feel any subjective or aggravation of HL along with new-onset tinnitus: all of the distress caused by tinnitus is not explained by its psychoacoustic characteristics [9,10], and chronic tinnitus (CT) does not necessarily involve pathophysiology of the auditory system [11]. Furthermore, evidence suggests that in addition to the initial pathology, the neural activity responsible for generating tinnitus involves neurocognitive and neuro-emotional networks as well as abnormal interactions between somatosensory, sensorimotor, and visual-motor systems [12]. For example, attentional and emotional states can be involved in the emergence and maintenance of tinnitus via top-down mechanisms [13]. 

Starting with the theoretical ‘*psychological model of tinnitus’* proposed by Hallam et al. [14], who observed that psychological factors play a role in the manifestation of tinnitus symptoms, a large body of literature now suggests that psychological variables are critical in tinnitus perception and distress [15]. Moreover, tinnitus is also associated with poorer performance across various broad cognitive domains [16]. Furthermore, certain personality traits like neuroticism and psychological disorders, especially anxiety and depression, often coexist with tinnitus and may act as predictors for tinnitus severity [17,18,19]; see [20].

While recent years have seen remarkable progress in understanding this tinnitus heterogeneity [21], the risk factors for tinnitus, as well as the mechanisms of tinnitus generation and maintenance, still need to be better understood [22]. Further, the role of psychological factors in determining distress in patients has long been recognized and remains a central theme in researchers’ and clinicians’ views of tinnitus [23]. Continuing, from a physiological perspective, the ‘*neurophysiological model of tinnitus*’ proposed by Jastreboff [24], which is now widely accepted [25], suggests that in addition to all levels of auditory system pathways, many other brain systems play a crucial role in the onset of tinnitus. 

Merging the psychological and neurophysiological approaches, Anderson and McKenna [26] have proposed a ‘*cognitive model of tinnitus’*. According to the authors, tinnitus is likely to disrupt cognitive functioning, and there are some indications that those who have it show impaired capacity to accomplish specific cognitive tasks (e.g., [27,28]). 

Moreover, studies have shown a strong association between tinnitus, anxiety, depression, and somatic awareness, which may have a confounding influence on cognitive performance [29], affecting information processing [30,31,32]. Furthermore, studies suggested interferences of tinnitus with executive function, short and long-term memory, and processing speed [16]. Finally, self-report measures of tinnitus distress require conscious recollection of symptomatology, further highlighting how the role of cognition in tinnitus is difficult to ignore [26]. Tinnitus and cognition, indeed, are two inseparable constructs whose interaction can be explained through neuroimaging methods applied during cognitive tasks [25]. For example, electroencephalography (EEG) and magnetoencephalography (MEG) studies in tinnitus have highlighted several abnormalities regarding the background cerebral oscillation in various frequency bands, such as theta, delta, alpha, beta, or gamma [33]; although there are conflicting data, a general tendency is a concentration of abnormalities mainly over the temporo-parietal and frontal regions [34]. Additionally, in about 85% of all cases of CT [35], the tinnitus sound is perceived constantly; thus, resting-state functional measurements seem well suited to identify the neuronal correlates of tinnitus. Moreover, as tinnitus is mainly experienced during rest, alterations in the default mode network (DMN) could be associated with it. Studies indeed have confirmed that the limbic system and attentional resting state networks are more active in the presence of tinnitus and may explain why persistent tinnitus results in mental fatigue [36]. The DMN is one of the most stable networks in the resting state of the brain, including multiple brain regions such as the prefrontal lobe, posterior cingulate cortex, and parietal cortices [37,38]. Moreover, together with DMF, the salience network (SN) and the frontoparietal networks (FPNs) are often called ‘canonical’ [39], as their interactions play a role in almost all cognitive functions [40]. The SN has attracted much attention for its role in the detection of salient information in the environment and the subsequent redirection of attentional resources and autonomic processes to generate adaptive cognitive and homeostatic responses [41]. The FPN, like the central executive network (typically including the prefrontal cortex and posterior parietal cortex [42]), is crucial for maintaining and processing information in working memory, problem solving, and decision making. Its activation is relatively strongest during cognitive effort and negatively correlated with the DMN [40]. Finally, the auditory network (AN) is responsible for recognizing noise and speech [43]. Interestingly, changes in the AN, SN, DMN, and FPN have been shown in patients with tinnitus [44,45].

Having mentioned the concept of a ‘*brain network’*, we believe it is important here to point out that network science is a branch of study that focuses on complex networks including those in computer, economic, cognitive, social, biological, and semantic domains. Thanks to the study of networks, it is increasingly easy to understand the involvement of resting-state network interactions in brain disorders [46], including tinnitus. As a matter of fact, *functional connectivity*—statistical relationships between a pair of brain regions that covary or correlate over time—is aberrant in many brain disorders such as anxiety, depression, and ADHD [47].

Based on network science principles, as proposed by De Ridder et al. [44], each aspect of tinnitus could be the result of connectivity changes among networks: the lateral pathway (i.e., the auditory network) and another resting-state network, such as the SN (suffering), the DMN (embodiment), the central executive network (cognitive disability), and motor network (physical disability). Neurocognitive network dynamics are often discussed in terms of rhythmic activity in different frequency ranges: delta (δ, 1–3.5 Hz), theta (θ, 4–7.5 Hz), alpha (α, 8–12 Hz), beta (β, 13–30 Hz), and gamma (γ, above 30 Hz) [48]. Moreover, EEG investigations have been shown as valid measures for the objective detection of tinnitus in several investigations [49,50] (for a recent overview, see [51]) and also for neurophysiological investigations during auditory cognitive tasks in groups of adult patients and children with sensory impairments [52,53,54,55,56]. Furthermore, spectral band analysis of the EEG offers a reliable and useful approach to understanding different psychiatric disorders. The literature reported differences in bands of the EEG power spectrum between controls and those with various psychiatric disorders, including depression, anxiety, and addiction [57]. Specifically, higher beta band powers were observed in anxious adults [58], and an excess of beta waves is associated with stress, anxiety, overthinking, and overstimulation [59]. Moreover, variability in human resting state EEG may reflect emotion regulation processes [60], while during cognitive tasks, an increase in the amplitude of the beta band was associated with the anxiety level of an individual [61].

Focusing more on the beta band in specific brain areas, studies have found increased frontal activity correlated with vigilance [62,63], while in the parietal brain region, beta activity is correlated with stress states [64,65] and tends to increase due to psychosocial stress in the reactive and recovery phase [66,67]. Moreover, enhanced beta activity in posterior brain areas has been associated with increased anxiety in adults [68]. From what has been introduced so far, audiology, neurobiology, and psychology are the disciplines generally conceptualizing the mechanisms of CT. Furthermore, concerning the mechanisms of onset or generation of tinnitus sensation, the functionality and dysfunctionality of the ear remain the dominant theme in both clinical practice and research. [69].

Advances in neuroimaging have highlighted the neuroplasticity of auditory and nonauditory brain regions, particularly emotion, attention, and memory regions, solidifying neuroscientists’ interest in this condition [70,71]. In addition to neurobiological evidence suggesting the role of psychological functions, patients often describe a significant negative impact of CT on daily life. This influence has sparked increasing interest in the contribution of psychological functioning to the tinnitus experience, beyond the co-morbidity or symptom of persistent sensation perception but as a potential contributing factor to ongoing awareness, volume, and severity of tinnitus sensation [23]. Recent reviews have addressed aspects of auditory and neurobiological functioning in CT [71,72,73] so, as Trevis et al. [32] suggest, a comprehensive review of the role of psychological functioning in this clinical population is now needed. Indeed, although mechanisms of tinnitus are investigated with network science and conceived about distinct disciplines such as audiology, biology, psychology, and physiology, no study to date has attempted to offer a multidisciplinary integrated neurophysiological, psychological, and audiological approach in an ecological in vivo experimental setting. Although numerous neuroscientific techniques have been used to investigate the pathology of tinnitus at the brain level ([74] for a review) and several paradigms have been applied to assess neural correlates of tinnitus [75], only a few experimental paradigms [76] have attempted to recreate the everyday ecological context of the tinnitus-perceiving patient.

Therefore, the aim of our study, through the use of a cutting-edge light EEG system, already employed in a previous study on tinnitus patients [76], is to investigate differences in psychological and electroencephalographic (beta band) variables and the presence of neuro-psycho-audio-physiological frontoparietal beta EEG networks in typical hearing patients with tinnitus (and in healthy controls) during a simple audio cognitive task in different levels of listening difficulties, and at resting state.

## 2. Materials and Methods 

This study was conducted at the Head and Neck Department of Polyclinic Umberto I Sapienza University of Rome, Italy. Informed consent was obtained from all participants involved in the study.

### 2.1. Experimental Sample

In this study, 19 participants were recruited: 12 chronic tinnitus (TIN) patients (7F, 5M; mean age ± SD: 47.416 ± 12.770) and 7 healthy control (CONT) participants (4F, 3M; mean age ± SD: 46.314 ± 16.331) (see Table 1 for participants details). The patients were recruited from the ENT ambulatory DAI Head and Neck of Polyclinic Umberto I of Rome, Italy.

The inclusion criterion for the TIN group was the perception of the primary symptom of tinnitus (unilateral and/or bilateral) for at least 3 months; no therapy (pharmacological and/or psychological) in place to treat tinnitus symptoms. The inclusion criteria common to all participants were as follows: normal hearing as assessed by pure-tone audiometry (PTA) tests averaging from 125 at 8 kHz down to 20 dB, and absence of diagnosed relevant medical conditions (e.g., psychiatric or neurological disorders) or anatomo-functional alterations that could impact the study, absence of taking psychoactive medications.

### 2.2. Audiological Assessment 

Patients completed two questionnaires to measure tinnitus severity: the Tinnitus Handicap Inventory (THI) [77] validated in Italian [78] and the Italian Tinnitus Questionnaire 12-item short form (TQ12-I) [79]. Moreover, we proposed a questionnaire to assess hyperacusis [80] defined as unusual tolerance to ordinary environmental sounds [81] to evaluate the possible impact on the clinical group considering that hyperacusis seems to increase in extent at times of anxiety, tiredness, or stress [82,83].

The THI is a 25-item tool with 3 response options (no, sometimes, yes) and a score range of 0 to 100. It was created to measure the functional and psychosocial effects of tinnitus and how it affects day-to-day living. The THI provides additional data to the traditional psychoacoustic assessment (e.g., pitch and loudness matching, minimum masking levels, residual inhibition). Tinnitus severity is categorized as follows: no handicap (0–16 points), ‘mild’ (18–36), ‘moderate’ (38–56), ‘severe’ (58–76), or ‘catastrophic’ tinnitus handicap (78–100 points). The THI has been used in neurophysiological studies and is widely recommended as a research tool for rating the severity of tinnitus [84]. 

The TQ12-I is the short form of the original 20-item test by the authors of [85] that allows the assessment of tinnitus-related distress. According to the TQ12-I, the grade of tinnitus distress is categorized as follows: no clinically relevant tinnitus distress (1–7 points); moderately distressed (8–12 points); severely distressed (13–18 points); and most severely distressed (>19 points).

The Hyperacusis Questionnaire validated in Italian [86] makes it possible to investigate clinical hyperacusis. The questionnaire is divided into two sections. The first consists of three binary questions that provide general information on noise exposure and auditory disorders. A total of 14 self-rating items make up the second section. They will be scored over 3 main dimensions: attentional (questions 1–4), social (questions 5–10), and emotional (questions 11–14). Each question/item has a 4-point scoring system: ‘no’ (0 points), ‘yes a little’ (1 point), ‘yes, a lot’ (2 points), or ‘yes, quite a lot’ (3 points). The Hyperacusis Questionnaire exhibits high levels of sensitivity in discriminating subjects with hyperacusis in the general population. A mean score greater than 28 is considered indicative of hyperacusis. Finally, through an interview conducted by a specialized health team (audiologist, psychologist), it was possible to collect specific information related to tinnitus symptomatology, such as the onset and duration of tinnitus, the lateralization, and the kind of sound perceived (Table 1).

### 2.3. Psychological Assessment 

Psychopathological symptoms in all participants were evaluated by the Italian version [87] of the Symptoms Checklist-90-Revised (SCL-90-R) [88], already used in studies with patients with tinnitus [89]. The SCL-90-R includes several different subscales exploring the severity of respondents’ symptoms over the previous seven days. Each item is rated on a 5-point Likert scale ranging from ‘Not at all’ (0) to ‘Extremely’. The checklist consists of nine subscales and three global indexes of distress. Somatization (SOM); Obsessive-compulsive (O-C); Interpersonal sensitivity (I-S); Depression (DEP); Anxiety (ANX); Hostility (HOS); Phobic anxiety (PHOB); Paranoid ideation (PAR); and Psychoticism (PSY) are the nine subscales. Seven additional items (OTHER) explore disturbances in appetite and sleep. The general indexes are as follows: the Global Severity Index (GSI) is the single best indicator of the current level or depth of an individual’s disorder. The Positive Symptom Total (PST) reflects how many symptoms the respondent endorses. Additionally, the Positive Symptom Distress Index (PSDI) can be understood as a gauge of symptom intensity because it reflects the average degree of distress reported for the symptoms endorsed; as such, it can be interpreted as a measure of symptom intensity.

Anxiety symptoms were evaluated with the STAI-Y questionnaire [90] for Italian adaptation, see [91], which separately assesses both state anxiety and trait anxiety. Each type of anxiety has its own scale of 20 scored questions. Participants answered 40 items on a 4-point Likert scale ranging from 0 (not at all) to 4 (very much so). The range score for each scale is 20–80, with the higher scores indicating greater anxiety. The STAI-Y is an instrument widely used in studies in the hearing field [92,93] and in EEG studies with tinnitus patients [11]. Spielberg proposed a categorization method dividing anxiety into trait anxiety and state anxiety based on how long the anxiety lasted [94]. State anxiety is a subjective feeling brought on by tension and other factors. It is characterized by a short duration and a certain intensity of physiological response. Individuals with high trait anxiety have cognitive biases that lead them to exaggerate the threat of external information or stimuli [95]. People with high trait anxiety are thought to be more vulnerable to anxiety disorders because of the strong behavioral, anatomical, and functional similarities between them and those with anxiety disorders [96].

The personality traits were assessed using the Italian version [97] of the Big Five Inventory 10-items (BFI-10) [98], a questionnaire developed based on the 44-item Big Five Inventory used in tinnitus studies (see [19]). The BFI-10 assesses the following personality traits: agreeableness/antagonism, conscientiousness/lack of direction, emotional stability/neuroticism, extraversion/introversion, and openness/closedness to experience. A five-point Likert scale is used to rate the items ranging from one = ‘disagree strongly’ to five = ’agree strongly’.

### 2.4. Electroencephalographic Assessment 

During the audio cognitive task, each participant wore the Mindtooth Touch EEG headset (https://www.mindtooth-eeg.com/) (accessed on 24 May 2024), which has already been used for the assessment of psychophysiological variables in cognitive neuroscience protocols [63], with 8 electrodes, placed, according to the 10–20 system [99] in prefrontal (AFz, AF3, AF4, AF7, AF8) and parietal (Pz, P3, P4) cortices plus ground (left mastoid) and reference (right mastoid). The EEG signal was first band-pass filtered with a fifth-order Butterworth filter at 2–30 Hz intervals. The blink artifacts were detected by employing the Reblinca method [100]. At a sampling frequency of 125 Hz, raw data were recorded through Mindtooth proprietary software (v.5.22) running on a laptop. Offline, the EEG signal was segmented into 1 s long epochs with 0.5 s of overlap to avoid any ‘boundary effect’. Then, we applied dedicated algorithms of the EEGLAB toolbox [101] to remove other sources of artifacts. In detail, the blink-free signal was divided into 1 s long epochs, and a threshold criterion was applied, i.e., the epochs with a signal amplitude exceeding ±80 mV (threshold) were labeled as ‘artifacts’ [102]. In the end, the EEG epochs marked as ‘artifacts’ were removed from the EEG dataset to have a clean EEG signal to perform the analyses. Finally, the Global Field Power (GFP) was calculated from the artifact-free EEG with a focus on the frequency band of interest focal for the aim of this study, Alpha and Beta over the *frontal* area of interest (AOI), channels AFz, AF3, AF4, AF7, and AF8 and the *parietal* AOI (channels Pz, P3, P4). These bands were defined accordingly with the Individual Alpha Frequency (IAF) value. Specifically, the IAF corresponds to the peak in the alpha band (typical IAF value is 10 Hz) obtained from the power spectrum of individual EEG signals over parietal sites during a rest condition [103], so we estimated it specifically from each subject through 1 minute of eyes closed, which was recorded before starting the experiment. Therefore, frequency bands were determined individually for each participant by using the IAF as the cutoff point between the lower and upper alpha band: alpha (IAF − 2 ÷ IAF + 2), and beta (IAF + 2 ÷ IAF + 16). The GFP was chosen because it describes brain EEG activity with the advantage of representing, in the time domain, the degree of synchronization of a specific cortical region of interest in a specific frequency band [104,105].

### 2.5. Audio Cognitive Task 

**Figure 1:**
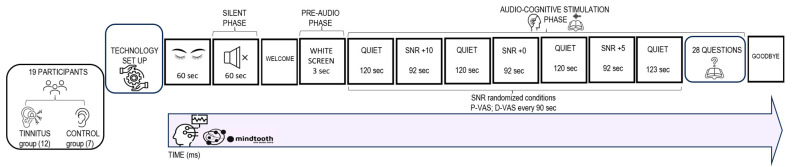
Graphical representation of the experimental protocol employed.

In the present study, we examined, through an integrated approach, the neurophysiological (EEG), behavioral, and cognitive mechanisms of tinnitus during a cognitive ecological listening task under varying stress conditions and in silence (resting state). The task lasted approximately 20 min and it consisted of 2 phases: the ‘silent phase’ and the ‘audio cognitive stimulation phase’ (see Figure 1 for task structure schematization). 

During the ‘*silent phase’*, the participants were asked to be relaxed and fix on for 60 s a point on the white screen in front without any exposure to external auditory stimuli. 

The ‘*audio-cognitive stimulation phase’* consisted of listening to a short story in Italian ‘*Storia di Gianna e delle sue chiavi’* recorded by a female voice, taken from the ‘*Progetto Babele Rivista Letteraria’* already used in neuroscientific experiments [76,106]. The total duration of audio stimulation was 11 min 39 s in 3 randomized signal-to-noise ratio (SNR) conditions (‘*noise conditions’*): +5; +10; 0 with an average duration of 1 min 31 s. Moreover, a ‘*quiet condition’* was presented at the beginning and the end of stimulation (average duration of 2 min 30 s each).

The stimulus was delivered by 2 audio speakers placed 1 m in front of the participants at face height, as in previous auditory neuroscience clinical studies [107]. Total auditory stimulation was set at 65 dB [56]. Before the start of auditory stimulation, participants were shown a blank screen for 3 s (*pre-audio phase*). The audio track was processed under various SNR conditions with the support of Audacity software (version 3.0.0) “Babble noise” [108] was the noise used, which has previously been employed to build experimental protocols in auditory neuroscience in samples of normal hearing and hearing-impaired persons [53,109]. 

During the ‘*audio-cognitive stimulation phase’*, the participant was asked to indicate at regular intervals of 90 s, for a total of 7 times, corresponding to the 7 Quiet and SNR conditions, on 2 distinct VASs already used for tinnitus patients [110] with a score from 0 to 100, the subjective perception of pleasantness and perceived difficulty during listening (self-reported data). At the end of the story, to investigate mnestic and attentional skills, the participant was asked to answer 28 multiple-choice questions about the content of the story (cognitive performance data). The audiobook presentation was controlled and displayed on a Lenovo PC with a 1024 × 768 monitor. The E-Prime software package (Psychology Software Tools, Pittsburgh, PA, USA, version 3.0) was used to collect the participants’ responses.

### 2.6. Statistical Analysis

Inferential statistical analyses were performed on the psychological and audiological questionnaires, neurophysiological measures, and self-reported and cognitive performance data. For all cases, the Shapiro–Wilk test of normality [111] demonstrated that most of the distributions of data were Gaussian; however, given the small number of experimental samples, non-parametric tests were employed in the analyses [112], with a significance of *p* = 0.05 used for testing for each dependent variables’ differences between the two groups (TIN; CONT). The psychological dependent variables considered were scores for each scale of the SCL-90-R; BFI-10; and STAI-Y questionnaires. The audiological dependent variables considered were the total score of the THI; TQ-I; and Hyperacusis tests. Beta EEG activity in the frontal and parietal AOIs for each auditory stimulation phase (SNRs: Quiet/+5/+10/+0) and at resting state (RS-silent phase) were the neurophysiological variables. Finally, we analyzed self-reported variables (pleasantness and difficulty) and cognitive performance variables (correct responses to the final questionnaire). A nonparametric Spearman’s correlation coefficient rho (ρ) was applied to assess the association between variables within each group. To investigate the significant linear relationships between variables and distinguish the influence independent from the interaction of dependent variables [113], we conducted a simple linear regression analysis of the correlated variables for each group. In fact, if two variables are highly correlated, it is then feasible to predict the value of one (the dependent variable) from the value of the other (the independent variable) using regression techniques [114]. Statistical analyses were performed using the computer software JASP (Version 0.17.2.1).

## 3. Results

### 3.1. Differences between Groups 

**Figure 2:**
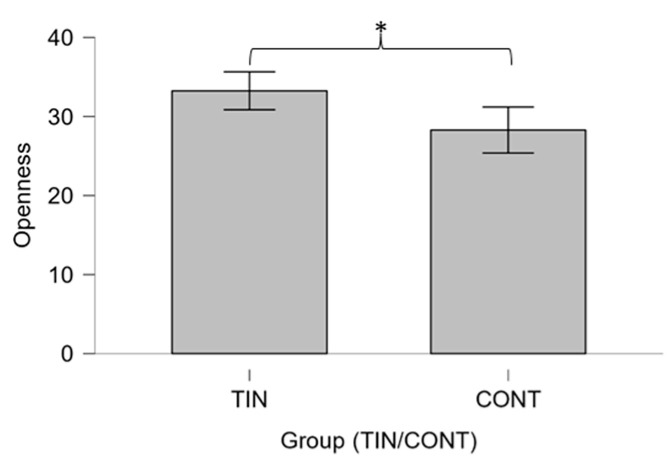
Box plot showing the comparison of Openness scale scores according to Big Five Inventory 10-items (BFI-10) between groups TIN (tinnitus) and CONT (controls) performed using the Mann–Whitney U-test. Significant differences between groups emerged (* *p* ≤ 0.05).

Statistical analysis showed no significant differences between the two groups for STAI-Y questionnaire scores (*p* > 0.05). Regarding the Big Five questionnaire, the MW test shows that TINNs have significantly higher scores for the ‘openness to experience’ trait than controls (U = 14.00, *p* = 0.019) (Figure 2). 

**Figure 3:**
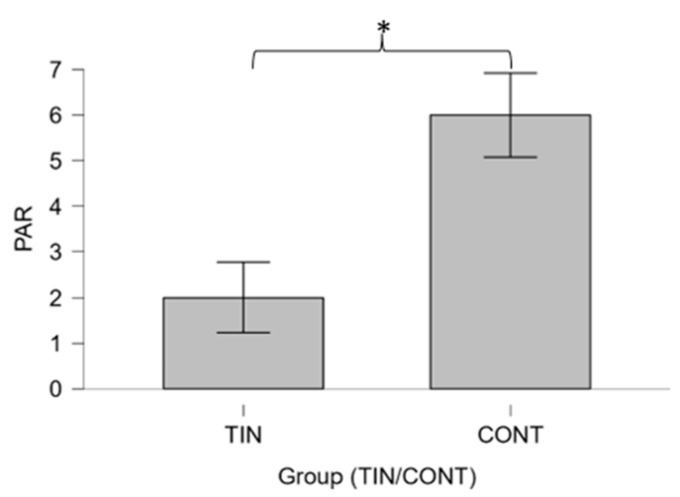
Boxplot showing the comparison between paranoid ideation (PAR) scale scores according to the SCL-90-R questionnaire between groups TIN (tinnitus) and CONT (controls) performed using the Mann–Whitney U-test. Significant differences between groups emerged (* *p* ≤ 0.05).

Furthermore, the TIN group showed significantly lower scores on the paranoid ideation SCL-90-R scale than the CONT group (U = 72.00; *p* = 0.011) (Figure 3).

No significant differences in neurophysiological variables emerge between the 2 groups (*p* > 0.05).

### 3.2. Correlation Results

#### 3.2.1. Psychological and Audiological Data

**Figure 4:**
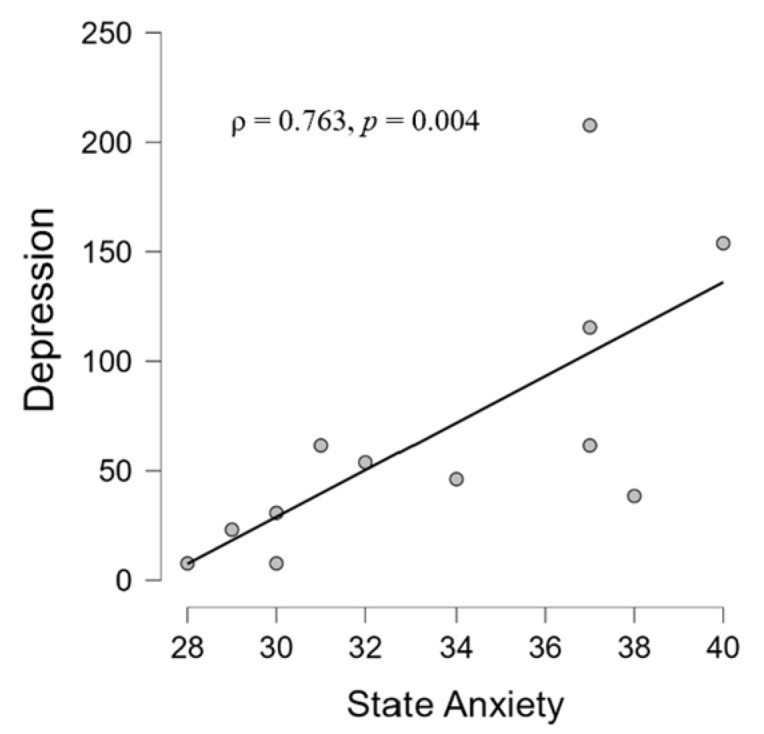
Scatter plot showing a positive correlation in the TIN group between state anxiety scales and depression scores according to the STAI-Y and SCL-90-R questionnaires, respectively.

Concerning the correlations between the SCL-90-R scales; STAI-Y scales; and BFI 10 scales, we found significant relationships between state anxiety and depression (ρ = 0.763, *p* = 0.004) in the TIN group (Figure 4). Regarding the relationships between the audiological variables and the psychometric scales, no significant correlation emerged (*p* > 0.05).

**Figure 5:**
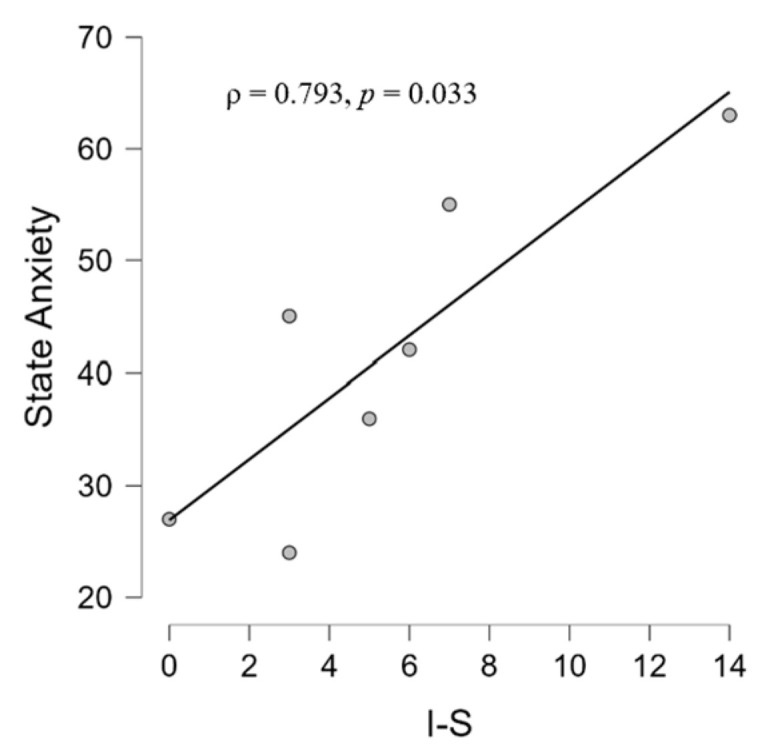
Scatter plot showing in the CONT group a positive correlation between interpersonal sensitivity (I-S) scale and state anxiety scale scores according to STAI-Y and SCL-90-R questionnaires, respectively.

**Figure 6:**
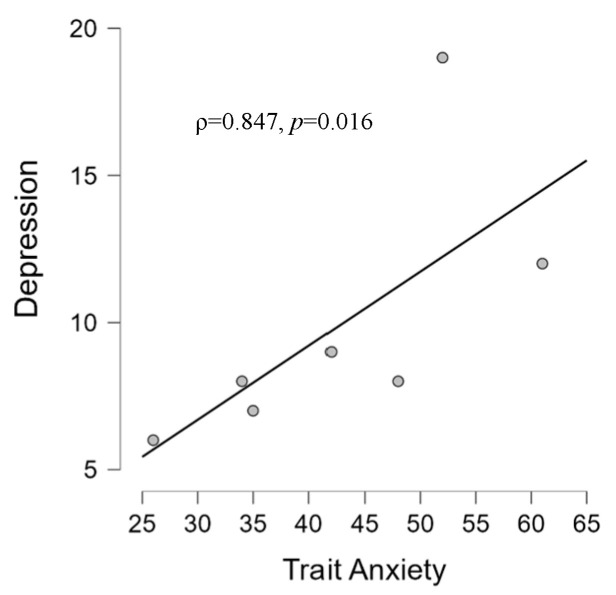
Scatter plot showing in the CONT group a positive correlation between depression scale and trait anxiety scale scores according to STAI-Y and SCL-90-R questionnaires, respectively.

For the CONT group, we found a significant relationship between state anxiety and Interpersonal sensibility (ρ = 0.793, *p* = 0.033) (Figure 5), and between trait anxiety and depression (ρ = 0.847, *p* = 0.016) (Figure 6).

#### 3.2.2. EEG, Psychological, and Audiological Data

**Figure 7:**
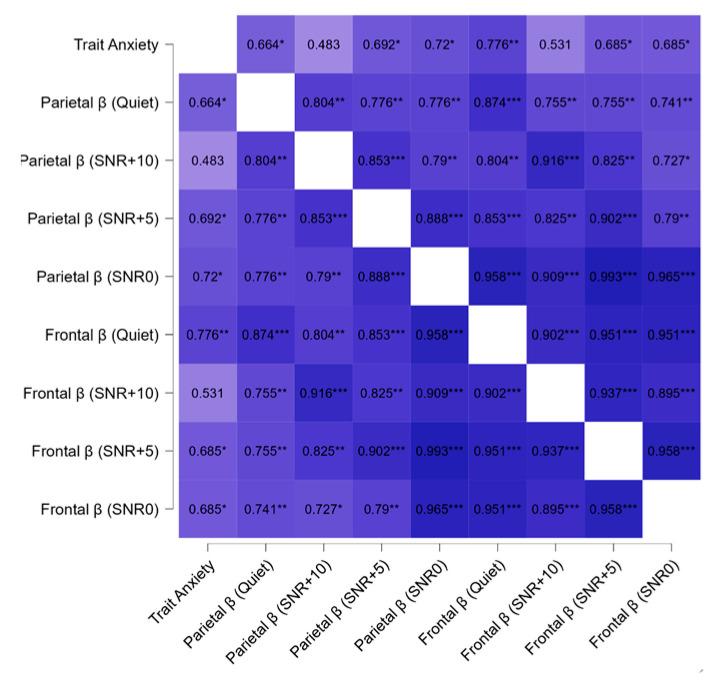
Heatmap of correlation coefficient among trait anxiety scores and beta (β) activity in frontal and parietal area during audio cognitive stimulation phase at different SNR levels (quiet, +5, +10, +0) in the TIN group. Spearman’s correlations (* *p* ≤ 0.05; ** *p* ≤ 0.01; *** *p* ≤ 0.001) are shown between the variables described.

During the ‘audio-cognitive stimulation phase’, regarding correlations within the TIN group among EEG and psychological and audiological variables, we found a positive correlation between beta activity in frontal and parietal areas in all noise conditions and correlations between trait anxiety and parietal and frontal beta in all conditions except during the condition SNR+10 (Figure 7). No correlation was significant in the control group.

**Figure 8:**
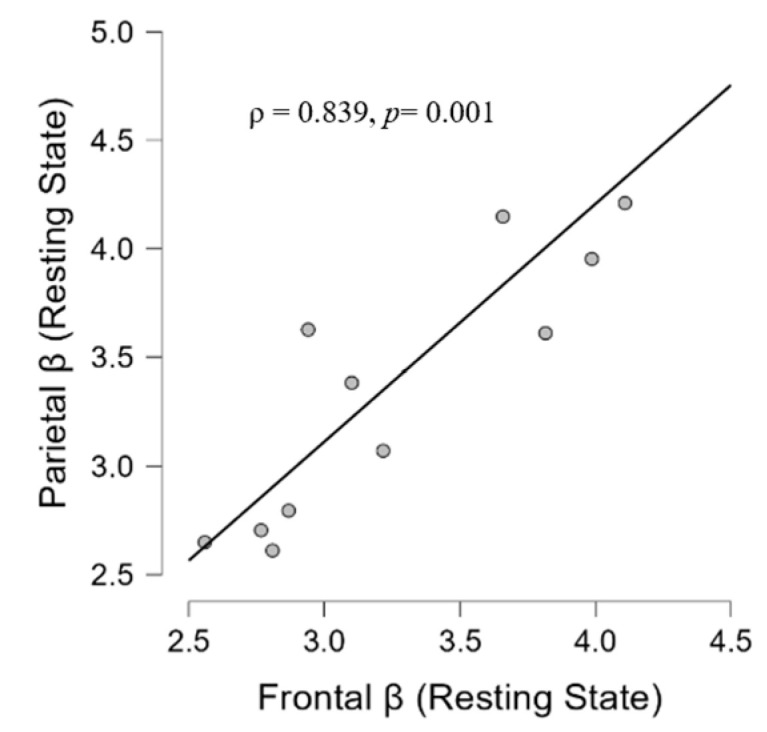
Scatter plot showing in the TIN group a positive correlation of beta activity between frontal and parietal area at resting state.

**Figure 9:**
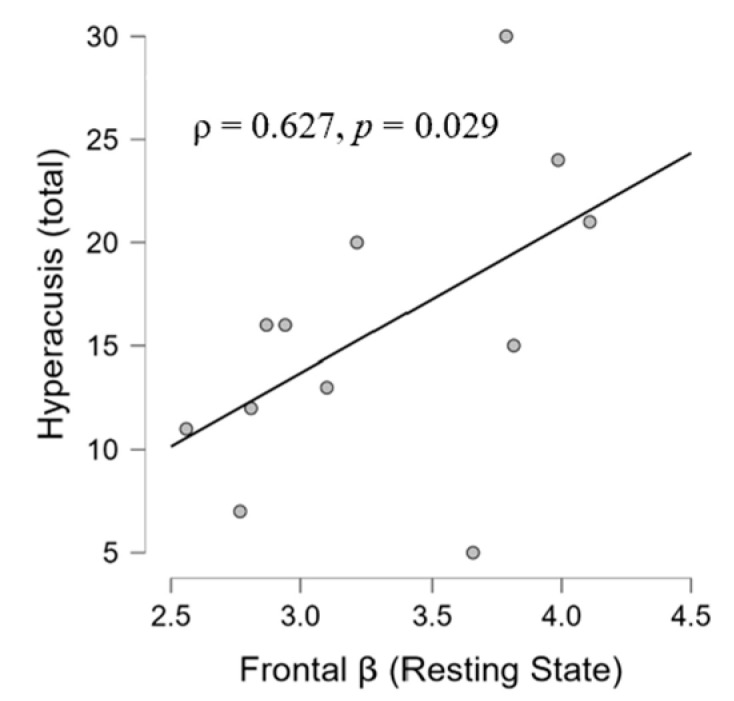
Scatter plot showing a positive correlation in the TIN group between beta activity in the frontal area at resting state and hyperacusis scores according to Khalfa’s questionnaire.

**Figure 10:**
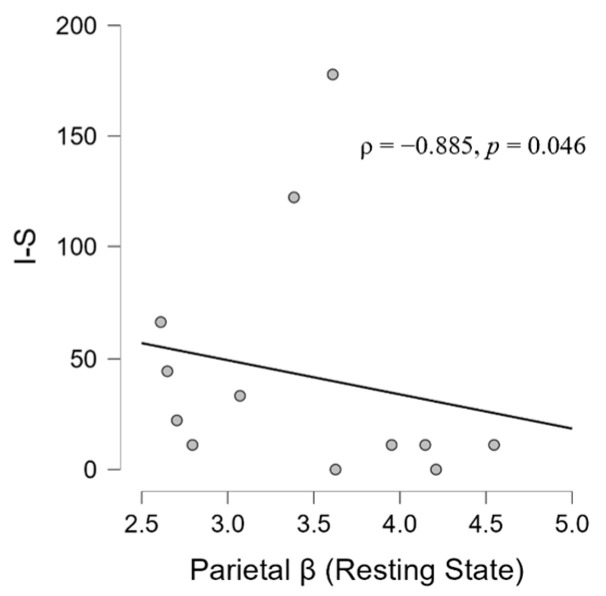
Scatter plot showing a negative correlation in the TIN group between beta activity in the parietal area and interpersonal sensitivity (I-S) scale scores according to the SCL-90-R questionnaire.

**Figure 11:**
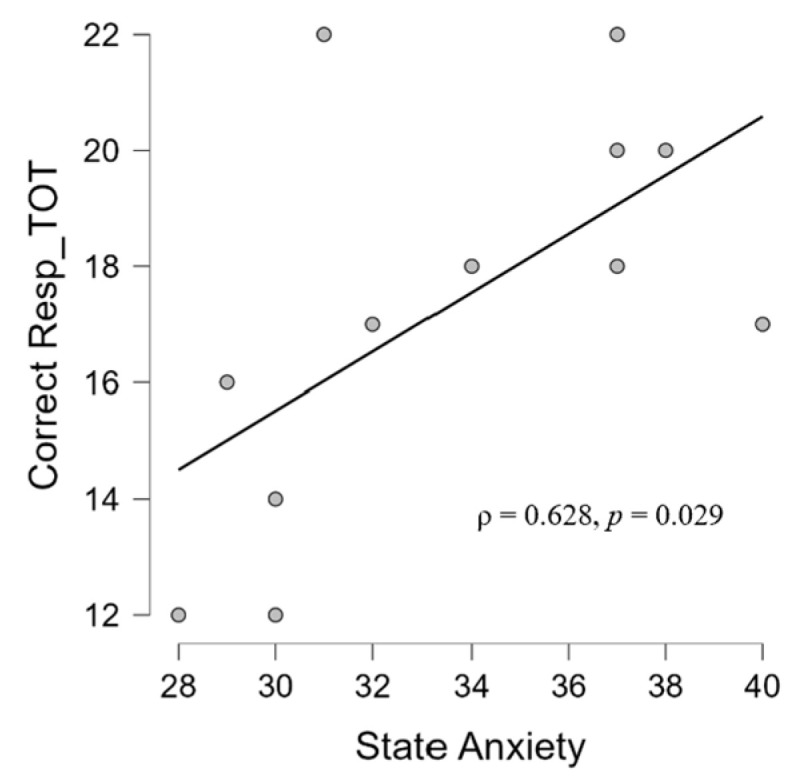
Scatter plot showing a positive correlation in TIN group between state anxiety scale score according to STAI-Y questionnaire and correct responses at the final questionnaire about the audiobook.

At the resting state during the ‘silent phase’, in the TIN group, we found a positive correlation between the frontal beta and parietal beta (ρ = 0.839, *p* = 0.001) (Figure 8). Moreover, the analysis has shown significant associations between frontal beta activity and hyperacusis in the resting state (ρ = 0.627, *p* = 0.029) (Figure 9) and between the parietal beta and I-S (ρ = −0.885, *p* = 0.046) (Figure 10). Moreover, a significant correlation also emerged between state anxiety and correct responses (ρ = 0.685, *p* = 0.046) (Figure 11). No correlation was significant in the CONT group.

### 3.3. Simple Linear Regression Models

Simple linear regression models were fitted to investigate the relationship between the audiological, psychological, and neurophysiological variables within TIN and CONT groups. In the CONT group, no significant results emerged. In the TIN group, concerning trait anxiety and the frontal beta during the ‘silent phase’, a significant regression was found (F (1,10) = 6.366, *p* = 0.03). The R^2^ was 0.388, indicating that the trait anxiety score explained approximately 39% of the variance in frontal beta activity at resting state. Moreover, between the two anxiety scales, we found a significant regression (F (1, 10) = 4.968, *p* = 0.05). The R^2^ was 0.332, indicating that trait anxiety explained approximately 33% of the variance of state anxiety scores. Finally, concerning frontal beta activity at resting state and hyperacusis, a regression approaching the borderline significance was found (F (1, 10) = 4.082, *p* = 0.071), with an R^2^ of 0.29 and an almost 30% possible contribution of beta frontal activation to hyperacusis. 

**Figure 12:**
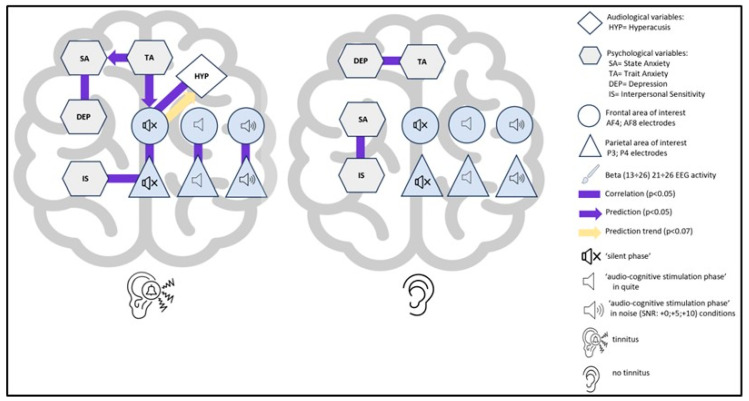
Schematic representation of the significant interconnections between the variables investigated (electroencephalographic, psychological, audiological) in the TIN (left) and CONT (right) groups.

Globally, a synthetic representation of the statistically significant relationships found between the investigated variables in TIN and CONT is given in Figure 12.

## 4. Discussion

### 4.1. Differences between Patients and Controls on Psychological Variables

Concerning the differences between the two groups, it is observed that the TIN group has a significantly higher openness personality trait (Figure 2). This result could be partially in line with Kleinstäuber et al.’s study [115] observing that high traits of openness to experience are correlated with better outcomes in tinnitus treatments. Thus, from the perspective of treatment choice, for the patients in our study, higher levels of openness could be a moderator to cognitive behavioral treatment outcomes. In addition, we would have expected more neuroticism in the TIN in line with the previous literature [116]. Not having any diagnosed severe tinnitus patients with hearing loss meant this association was probably not verifiable. Surely in a larger sample (both patients and controls), more differences could be observed.

Personality traits were not associated with pathology in our sample, contrary to the findings of some studies (see [19]). In 2014, the authors of [117] analyzed the relationship between tinnitus and personality by reviewing the literature from 1968 to 2012, finding an association between tinnitus and trait neuroticism. However, the studies considered in the cited review assessed personality traits with a different diagnostic tool than the one used in the present study. Instead, our result seems to be in line with what the authors of [118] stated: that (high) THI scores did not predict high scores on any of the personality traits.

Regarding the higher scores in paranoid ideation found in CONT rather than in TIN (Figure 3) (of slight clinical significance, however), one could speculate that the result was due to the characteristics of the participants in the control group, who were not selected on the basis of SCL-90-R scale values but on the basis of the absence of previous psychiatric diagnoses and symptoms of tinnitus and hearing disorders. Furthermore, as the SCL-90-R is a very long (90 items) psychometric Likert-type questionnaire that assesses the presence of numerous psychological symptoms, the higher values obtained by the CONT group (unaccustomed to psychological assessments) could be due to doubts, disorientation in the specific questions asked, and bias due to the Likert scale [119].

No significant differences emerged between the two groups on the anxiety STAI-Y questionnaire in contrast to other evidence showing that subjects with tinnitus have higher anxiety [20]. This could be due to the fact that no patients manifest severe tinnitus (Table 1). However, in 11 studies of the 17 reviewed in [20], patients were over 65 years of age. As our sample was relatively young (47.416 ± 12.770), anxiety symptoms in tinnitus patients could be age related. However, further studies with generational comparisons are necessary to confirm this interpretation.

### 4.2. Correlations between Psychological and Audiological Variables

Concerning the correlations between psychological questionnaires, we observed different patterns between the two groups. Firstly, it should be clarified that interpersonal sensitivity (I-S) is the capacity and degree to which individuals react to other people’s behaviors and feelings, social interactions, and their environment [120]. Interpersonal sensitivity, then, is the degree to which people care intemperately about their interpersonal relationships and fear being rejected or criticized by others. The correlation (ρ = 0.793) between I-S and state anxiety found in CONT (Figure 5) is, therefore, in line with evidence showing that those with high interpersonal sensitivity have greater chances of suffering from deficient social interactions and interpersonal stress [121]. State anxiety in the clinical group, on the other hand, correlated with a depressive dimension (ρ = 0.763), which is in line with the literature that shows the presence of anxiety and depression in tinnitus patients [122] and, specifically, a correlation between anxiety and depression [123]. The fact that we did not find an association with the THI could be because, on average, the group experienced mild tinnitus (mean THI score = 20, see Table 1) whereas the association between trait anxiety (a stable propensity to experience anxiety, and tendencies to perceive stressful situations as threatening [93]) and depression in the CONT group (ρ = 0.847) could suggest trait anxiety as a vulnerability factor to depressive symptoms in the general population [124]. Finally, state anxiety is positively related to performance in patients (ρ = 0.628) (Figure 11), which is in contrast to studies showing a negative relationship between performance and anxiety [125] but in agreement with other studies showing a positive relationship between anxiety and performance [126]. It could be hypothesized that the performance-supportive anxiety of the task may be due to the presence of tinnitus inevitably leading to a higher state of neurophysiological activation than state anxiety, meaning, therefore, that state anxiety (remember, not pathological in our samples) in tinnitus patients performing an auditory cognitive task may be functional to the task. This hypothesis is part of those who agree that anxiety can be a multifaceted agent [127]: at low levels, it can be a reason, but at high levels, it can be an obstacle.

### 4.3. Differences between Patients and Controls on Beta Band Activity

Regarding the EEG results, there were no significant differences in beta-band activity between the TIN and CON, contrary to what has been reported in the literature, neither during a listening task [128] nor during a resting state [129]. However, in [129], the clinical sample consisted of patients with tinnitus and hearing impairment whereas our experimental sample consisted of typical hearing patients. However, the clinical sample evaluated in [128] had clinical pathologies that we excluded.

### 4.4. Psycho-Audio-EEG Interconnections 

Regarding the TIN group, the beta-band cortical statistically significant interconnections in different noise conditions (Figure 7) and at resting state in silence (Figure 8) could be dependent on tinnitus as high-frequency waves have been shown to be associated with the volume of the tinnitus [130]. Furthermore, the fronto-parietal associations’ power changed as listening difficulty varies (Figure 7) and this could suggest the ‘physiological’ intensification of tinnitus. Notably, these associations were significant except in the +10 SNR condition, probably because it is the most ‘ecological’ listening condition. Furthermore, these results seem to corroborate the counterintuitive phenomenon of stochastic resonance (SR), according to which an optimal amount of noise can, under certain circumstances, be beneficial for cognitive performance [131], as already found in previous studies by our group [54].

We feel it is important to emphasize that the association of beta activity between the frontal and parietal AOI was also present during the ‘silent phase’ (ρ = 0.839) (Figure 8), which is in line with source EEG studies with tinnitus patients. Specifically, the authors of [129] showed a generalized power increase in delta and theta limited to fronto-centro-parietal sites in the beta domain, while in a resting EEG study of patients with severe tinnitus as compared to healthy controls a significant increase in Z-score power over the frequency ranges from 0.5 to 22 Hz was reported, which was dominant in fronto-temporal electrodes [132]. The significant fronto-parietal beta association that emerged from our analysis could signify a marker not strictly related to the auditory task in patients, identifying a possible stable neurophysiological network in tinnitus. Considering that beta activity in frontal cortices has been observed to be associated with vigilance states [63], its correlation with the parietal AOI could indicate a neurophysiological state of persistent fronto-parietal activation in tinnitus patients.

Furthermore, the positive correlation we observe between beta activity in the frontal areas and hyperacusis (ρ = 0.627) (Figure 9) could decline into an auditory-linked neurophysiological vigilance. This hypothesis is in line with studies showing that hyperacusis is accompanied by an increase in beta activity [133] and is supported by the role of the beta band in alert state cognitive functions and decision making [134,135,136]. Furthermore, our result confirms the associations between tinnitus and hyperacusis [4] despite the fact that Khalfa’s questionnaire was not significantly correlated with the THI (this could be because of the moderate level of tinnitus perceived by the participants, see Table 1, since the severity of tinnitus depends on the degree of deafness [86], and, here, we remember that the clinical sample was made up of normal hearing). So, the triple-correlational axis among hyperacusis and the frontal and parietal beta in different listening conditions suggests the presence of increased stress, attention/sensibility to external stimuli, and anxiety in the TIN.

This interpretative hypothesis of a prior neuro-psychophysiological status in tinnitus is further supported by the proposed regression model showing the significant prediction given by trait anxiety. Indeed, as in a psycho-neuro-audiological cascade (Figure 12) converging with evidence showing the impact of trait anxiety on frontal activation asymmetries [137], trait anxiety could impact, independently of the cognitive audio task, beta activity in the frontal area, which could be the (obviously, hypothetical) cause of hyperacusis in the patient, and also could be associated with parietal AOI activity. And further, because, as we have anticipated, an increase in beta signal has been associated with an increase in anxiety or a decrease in relaxation level (e.g., [138,139,140,141,142]). The evidence of this psycho-neuro-audiological cascade could be corroborated by the correlation between beta activity in parietal areas and I-S (ρ = −0.885) (Figure 10), a dimension considered a risk factor for anxiety [143]. Here, we may have obtained a neurophysiological pattern of this association. Moreover, our data suggest that trait anxiety would seem to predict state anxiety in patients that could, at certain levels, impair performance on cognitive tasks [125].

## 5. Conclusions

Starting from the evidence showing that many clinical aspects of tinnitus are still enigmatic and cannot be fully explained by current models [144], this study identified significant associations between psychological factors, EEG beta activity, and audiological variables in tinnitus patients. 

The findings support the presence of a neuro-psycho-audio tinnitus network (NPAT) characteristic of the patient. Also, we saw how anxious personality traits could be linked to beta brain activity over the frontal cortex, triggering possible associations with hyperacusis and parietal cortices. This evidence suggests that an integrated approach to treatment, combining cognitive behavioral therapy and neurofeedback (e.g., [145,146]), may be beneficial with respect to psychological and/or pharmacological therapy alone. 

## 6. Limitations

This study has its limitations, which nevertheless open up new experiments. First of all, the experimental sample should be enlarged in the future to obtain greater validity for the results. Moreover, to further investigate the associations between anxiety pathology—EEG—and audiological variables, it would be appropriate to include a sample with anxiety disorders and higher values on audiological questionnaires (THI; hyperacusis) to assess the differences.

## Figures and Tables

**Table 1:** Demographic and clinical data concerning 12 patients (TIN group) and demographic data concerning the 7 controls (CONT group). In particular, the table shows for each participant (P) gender; group (G); TIN (T); CONT (C); age; tinnitus onset; monolateral (M)/bilateral (B) tinnitus perception; qualitative description of perceived sound/noise; current situations of occurrence; activities (act.)/situations (sit.) that reduce the perception of tinnitus; if the participant had the perception of tinnitus during the audio cognitive task; Tinnitus Handicap Inventory (THI) score; grade of tinnitus severity in line with THI scores: mild (mil); moderate (mod); Italian Tinnitus Questionnaire 12-item short form (TQ-12) scores; Khalfa Hyperacusis questionnaire scores (HYP).

P	G	Age	Tinnitus Onset	M/B	Description of Perceived Sound/Noise	Current Situations of Occurrence	Tinnitus-Reducing act./sit.	Perception during the Task	THI	Tinnitus Severity	TQ-12	HYP
1	T	57.597	2 months ago	M	Loud high-pitched whistle	Before sleeping	Being with people; listening to music	yes	14	mil	8	13
2	T	59.526	5–6 years ago	B	Water brook	Always	Watching TV	yes	14	mil	7	7
3	T	47.715	2 years ago, being alone in the house in the night	B	High-pitched but slight whistling; sound ‘as of ambulance’.	In silence	Everyday life because he does street work	no	20	mod	11	15
4	T	26.926	End of June 2022	B	Ambulance sound; Tibetan bells; candy frizz	At work (switchboard operator in a pizzeria)	Nothing	no	32	mod	13	11
5	T	61.192	2 years ago, at the end of the lockdown for COVID-19	B	Pulsating whistle	Before sleeping	Listening to music; working and focusing attention on something	no	14	mil	4	30
6	T	47.096	First period in 2000, from 2019 strong perception	B	As of a power plan in operation; electrical circuit	Always	Nothing	no	48	mod	5	16
7	T	42.359	October 2022 in conjunction with strong emotional stress	M	Vigilant whistle.	Always	Nothing	no	26	mod	5	24
8	T	55.397	Post COVID-19 infection	B	It appeared as an itchy and muffled sensation, then turned into …	Always early in the morning and before sleep	During the day the tinnitus is not felt	no	18	mod	2	5
9	T	54.301	June 2022 after a severe cold	M	Ear plugged; violin string shrill	The evening before sleeping, in the silence	If distracted the patient reports feeling better	no	17	mod	9	16
10	T	58.444	2–3 years ago	B	Buzzing as a refrigerator	The evening before sleeping; watching TV	Distracted by cooking; by work	yes	15	mil	14	21
11	T	31.814	8–9 years ago, worked at airport on runways	B	Left whistle; bilateral pulsating sound very annoying	In the silence	Listening to music; playing PC with headphones	no	11	mil	8	12
12	T	26.630	2.5 years ago	B	Hissing, high frequency, like brake on rails	In the evening before falling asleep; tired	Sounds; white noise (use of dedicated APPs)	yes	12	mil	11	20
13	C	29.225	-	-	-	-	-	-	-	-	-	-
14	C	61.485	-	-	-	-	-	-	-	-	-	-
15	C	33.521	-	-	-	-	-	-	-	-	-	-
16	C	70.918	-	-	-	-	-	-	-	-	-	-
17	C	41.39	-	-	-	-	-	-	-	-	-	-
18	C	32.162	-	-	-	-	-	-	-	-	-	-
19	C	55.504	-	-	-	-	-	-	-	-	-	-

## Data Availability

The data presented in this study are available upon request from the corresponding author. The data are not publicly available because of ethical concerns.

## References

[1] Elgoyhen A.B., Langguth B., De Ridder D., Vanneste S. (2015). Tinnitus: Perspectives from human neuroimaging. Nat. Rev. Neurosci..

[2] Langguth B., Kreuzer P.M., Kleinjung T., De Ridder D. (2013). Tinnitus. Causes and clinical management. Lancet Neurol..

[3] De Ridder D., Schlee W., Vanneste S., Londero A., Weisz N., Kleinjung T., Shekhawat G.S., Elgoyhen A.B., Song J.-J., Andersson G. (2021). Tinnitus and tinnitus disorder: Theoretical and operational definitions (an international multidisciplinary proposal). Prog. Brain Res..

[4] Baguley D.M., Hoare D.J. (2018). Hyperacusis: Major research questions. HNO.

[5] Gibrin P.C., Melo J.J., Marchiori L.L. (2013). Prevalence of tinnitus complaints and probable association with hearing loss, diabetes mellitus and hypertension in elderly. Codas.

[6] Killion M. (2002). New thinking on hearing in noise: A generalized articulation index. Sem. Hear..

[7] Hennig T.R., Costa M.J., Urnau D., Becker K.T., Schuster L.C. (2011). recognition of speech of normal-hearing individuals with tinnitus and hyperacusis. Int. Arch. Otorhinolaryngol..

[8] Sahoo J.P. (2014). The effect of tinnitus on some psychoacoustical abilities in individuals with normal hearing sensitivity. Int. Tinnitus J..

[9] Scott B., Lindberg P., Lyttkens L., Melin L. (1990). Predictors of tinnitus discomfort, adaptation and subjective loudness. Br. J. Audiol..

[10] Park Y., Shin S.H., Byun S.W., Lee Z.Y., Lee H.Y. (2023). Audiological and psychological assessment of tinnitus patients with normal hearing. Front. Neurol..

[11] Czornik M., Malekshahi A., Mahmoud W., Wolpert S., Birbaumer N. (2022). Psychophysiological treatment of chronic tinnitus: A review. Clin. Psychol. Psychother..

[12] Cacace A.T. (2003). Expanding the biological basis of tinnitus: Crossmodal origins and the role of neuroplasticity. Hear. Res..

[13] Shore S.E., Roberts L.E., Langguth B. (2016). Maladaptive plasticity in tinnitus—Triggers, mechanisms and treatment. Nat. Rev. Neurol..

[14] Hallam R.S., Rachman S., Hinchcliffe R., Rachman S. (1984). Psychological aspects of tinnitus. Contributions to Medical Psychology.

[15] Durai M., Searchfield G. (2016). Anxiety and depression, personality traits relevant to tinnitus: A scoping review. Int. J. Audiol..

[16] Clarke N.A., Henshaw H., Akeroyd M.A., Adams B., Hoare D.J. (2020). Associations between subjective tinnitus and cognitive performance: Systematic review and meta-analyses. Trends Hear..

[17] Cronlein T., Langguth B., Pregler M., Kreuzer P.M., Wetter T.C., Schecklmann M. (2016). Insomnia in patients with chronic tinnitus: Cognitive and emotional distress as moderator variables. J. Psychosom. Res..

[18] Mazurek B., Böcking B., Dobel C., Rose M., Brüggemann P. (2023). Tinnitus and Influencing Comorbidities. Laryngorhinootologie.

[19] Van Munster J.J., Van der Valk W.H., Stegeman I., Lieftink A.F., Smit A.L. (2020). The relationship of tinnitus distress with personality traits: A systematic review. Front. Neurol..

[20] Yang D., Zhang D., Zhang X., Li X. (2024). Tinnitus-associated cognitive and psychological impairments: A comprehensive review meta-analysis. Front. Neurosci..

[21] Cederroth C.R., Gallus S., Hall D.A., Kleinjung T., Langguth B., Maruotti A., Meyer M., Norena A., Probst T., Pryss R.C. (2019). Editorial: Towards an Understanding of Tinnitus Heterogeneity. Front. Aging Neurosci..

[22] Baguley D., McFerran D., Hall D. (2013). Tinnitus. Lancet.

[23] McKenna L., Handscomb L., Hoare D.J., Hall D.A. (2014). A scientific cognitive-behavioral model of tinnitus: Novel conceptualizations of tinnitus distress. Front. Neurol..

[24] Jastreboff P.J. (1990). Phantom auditory perception (tinnitus): Mechanisms of generation and perception. Neurosci. Res..

[25] Czornik M., Birbaumer N., Braun C., Hautzinger M., Wolpert S., Löwenheim H., Malekshahi A. (2022). Neural substrates of tinnitus severity. Int. J. Psychophysiol..

[26] Andersson G., McKenna L. (2006). The role of cognition in tinnitus. Acta Oto-Laryngol..

[27] Hallam R.S., McKenna L., Shurlock L. (2004). Tinnitus impairs cognitive efficiency. Int. J. Audiol..

[28] Mohamad N., Hoare D.J., Hall D.A. (2016). The consequences of tinnitus and tinnitus severity on cognition: A review of the behavioural evidence. Hear. Res..

[29] Tegg-Quinn S., Bennett R.J., Eikelboom R.H., Baguley D.M. (2016). The impact of tinnitus upon cognition in adults: A systematic review. Int. J. Audiol..

[30] Broadbent D.E., Broadbent M.H., Jones J.L. (1986). Performance correlates of self-reported cognitive failure and of obsessionality. Br. J. Clin. Psychol..

[31] Andersson G., Bakhsh R., Johansson L., Kaldo V., Carlbring P. (2005). Stroop facilitation in tinnitus patients: An experiment conducted via the world wide web. CyberPsychol. Behav..

[32] Trevis K.J., McLachlan N.M., Wilson S.J. (2018). A systematic review and meta-analysis of psychological functioning in chronic tinnitus. Clin. Psychol. Rev..

[33] Houdayer E., Teggi R., Velikova S., Gonzalez-Rosa J.J., Bussi M., Comi G., Leocani L. (2015). Involvement of cortico-subcortical circuits in normoacousic chronic tinnitus: A source localization EEG study. Clin. Neurophysiol..

[34] Schlee W., Hartmann T., Langguth B., Weisz N. (2009). Abnormal resting-state cortical coupling in chronic tinnitus. BMC Neurosci..

[35] Schecklmann M., Landgrebe M., Langguth B. (2014). Phenotypic characteristics of hyperacusis in tinnitus. PLoS ONE.

[36] Schmidt S.A., Akrofi K., Carpenter-Thompson J.R., Husain F.T. (2013). Default mode, dorsal attention and auditory resting state networks exhibit differential functional connectivity in tinnitus and hearing loss. PLoS ONE.

[37] Greicius M.D., Krasnow B., Reiss A.L., Menon V. (2003). Functional connectivity in the resting brain: A network analysis of the default mode hypothesis. Proc. Natl. Acad. Sci. USA.

[38] Knyazev G.G., Slobodskoj-Plusnin J.Y., Bocharov A.V., Pylkova L.V. (2011). The default mode network and EEG alpha oscillations: An independent component analysis. Brain Res..

[39] Uddin L.Q., Betzel R.F., Cohen J.R., Damoiseaux J.S., De Brigard F., Eickhoff S.B., Fornito A., Gratton C., Gordon E.M., Laird A.R. (2023). Controversies and progress on standardization of large-scale brain network nomenclature. Netw. Neurosci..

[40] Schimmelpfennig J., Topczewski J., Zajkowski W., Jankowiak-Siuda K. (2023). The role of the salience network in cognitive and affective deficits. Front. Hum. Neurosci..

[41] Ince S., Steward T., Harrison B.J., Jamieson A.J., Davey C.G., Agathos J.A., Moffat B.A., Glarin R.K., Felmingham K.L. (2023). Subcortical contributions to salience network functioning during negative emotional processing. NeuroImage.

[42] Gong D., He H., Ma W., Liu D., Huang M., Dong L., Gong J., Li J., Luo C., Yao D. (2016). Functional integration between salience and central executive networks: A role for action video game experience. Neural Plast..

[43] Xiong B., Liu Z., Li J., Huang X., Yang J., Xu W., Chen Y.-C., Cai Y., Zheng Y. (2023). Abnormal Functional Connectivity within Default Mode Network and Salience Network Related to Tinnitus Severity. J. Assoc. Res. Otolaryngol..

[44] De Ridder D., Vanneste S., Song J.-J., Adhia D. (2022). Tinnitus and the triple network model: A perspective. Clin. Exp. Otorhinolaryngol..

[45] Husain F.T., Schmidt S.A. (2014). Using resting state functional connectivity to unravel networks of tinnitus. Hear. Res..

[46] Telesford Q.K., Simpson S.L., Burdette J.H., Hayasaka S., Laurienti P.J. (2011). The brain as a complex system: Using network science as a tool for understanding the brain. Brain Connect..

[47] Menon V. (2011). Large-scale brain networks and psychopathology: A unifying triple network model. Trends Cogn. Sci..

[48] Meehan T.P., Bressler S.L. (2012). Neurocognitive networks: Findings, models, and theory. Neurosci. Biobehav. Rev..

[49] Cartocci G., Attanasio G., Fattapposta F., Locuratolo N., Mannarelli D., Filipo R. (2012). An electrophysiological approach to tinnitus interpretation. Int. Tinnitus J..

[50] Adjamian P. (2014). The application of electro- and magneto-encephalography in tinnitus research—Methods and interpretations. Front. Neurol..

[51] Yasoda-Mohan A., Vanneste S., Schlee W., Langguth B., De Ridder D., Vanneste S., Kleinjung T., Møller A.R. (2024). The Electrophysiological Explorations in Tinnitus Over the Decades Using EEG and MEG. Textbook of Tinnitus.

[52] Inguscio B.M.S., Cartocci G., Sciaraffa N., Nicastri M., Giallini I., Aricò P., Greco A., Babiloni F., Mancini P. (2024). Two are better than one: Differences in cortical EEG patterns during auditory and visual verbal working memory processing between Unilateral and Bilateral Cochlear Implanted children. Hear. Res..

[53] Inguscio B.M.S., Cartocci G., Sciaraffa N., Nicastri M., Giallini I., Greco A., Babiloni F., Mancini P. (2022). Gamma-Band Modulation in Parietal Area as the Electroencephalographic Signature for Performance in Auditory–Verbal Working Memory: An Exploratory Pilot Study in Hearing and Unilateral Cochlear Implant Children. Brain Sci..

[54] Inguscio B.M.S., Mancini P., Greco A., Nicastri M., Giallini I., Leone C.A., Grassia R., Di Nardo W., Di Cesare T., Rossi F. (2022). ‘Musical effort’ and ‘musical pleasantness’: A pilot study on the neurophysiological correlates of classical music listening in adults normal hearing and unilateral cochlear implant users. Hear. Balance Commun..

[55] Cartocci G., Inguscio B.M.S., Giorgi A., Vozzi A., Leone C.A., Grassia R., Di Nardo W., Di Cesare T., Fetoni A.R., Freni F. (2023). Music in noise recognition: An EEG study of listening effort in cochlear implant users and normal hearing controls. PLoS ONE.

[56] Cartocci G., Giorgi A., Inguscio B.M.S., Scorpecci A., Giannantonio S., De Lucia A., Garofalo S., Grassia R., Leone C.A., Longo P. (2021). Higher right hemisphere gamma band lateralization and suggestion of a sensitive period for vocal auditory emotional stimuli recognition in unilateral cochlear implant children: An EEG study. Front. Neurosci..

[57] Newson J.J., Thiagarajan T.C. (2019). EEG frequency bands in psychiatric disorders: A review of resting state studies. Front. Hum. Neurosci..

[58] Pavlenko V.B., Chernyi S.V., Goubkina D.G. (2009). EEG Correlates of Anxiety and Emotional Stability in Adult Healthy Subjects. Neurophysiology.

[59] Engel A.K., Fries P. (2010). Beta-band oscillations—Signaling the status quo?. Curr. Opin. Neurobiol..

[60] Knyazev G.G. (2007). Motivation, emotion, and their inhibitory control mirrored in brain oscillations. Neurosci. Biobehav. Rev..

[61] Chaitanya M.N., Jayakkumar S., Chong E., Yeow C.H. A wearable, EEG-based massage headband for anxiety alleviation. Proceedings of the 2017 39th Annual International Conference of the IEEE Engineering in Medicine and Biology Society (EMBC).

[62] Molina E., Correa Á., Sanabria D., Jung T.P. Tonic EEG dynamics during psychomotor vigilance task. Proceedings of the 2013 6th International IEEE/EMBS Conference on Neural Engineering (NER).

[63] Sciaraffa N., Di Flumeri G., Germano D., Giorgi A., Di Florio A., Borghini G., Vozzi A., Ronca V., Varga R., van Gasteren M. (2022). Validation of a Light EEG-Based Measure for Real-Time Stress Monitoring during Realistic Driving. Brain Sci..

[64] Borghini G., Aricò P., Di Flumeri G., Babiloni F. (2017). Neurophysiological Signals Processing. Industrial Neuroscience in Aviation: Evaluation of Mental States in Aviation Personnel.

[65] Ronca V., Martinez-Levy A.C., Vozzi A., Giorgi A., Aricò P., Capotorto R., Borghini G., Babiloni F., Di Flumeri G. (2023). Wearable Technologies for Electrodermal and Cardiac Activity measurements: A Comparison between Fitbit Sense, Empatica E4 and Shimmer GSR3+. Sensors.

[66] Vanhollebeke G., De Smet S., De Raedt R., Baeken C., van Mierlo P., Vanderhasselt M.A. (2022). The neural correlates of psychosocial stress: A systematic review and meta-analysis of spectral analysis EEG studies. Neurobiol. Stress..

[67] Giannakakis G., Grigoriadis D., Giannakaki K., Simantiraki O., Roniotis A., Tsiknakis M. (2019). Review on psychological stress detection using biosignals. IEEE Trans. Affect. Comput..

[68] Shanok N.A., Jones N.A. (2024). EEG Asymmetry Characteristics in Relation to Childhood Anxiety Subtypes: A Dimensional Approach. Clin. EEG Neurosci..

[69] Eggermont J.J., Roberts L.E. (2004). The neuroscience of tinnitus. Trends Neurosci..

[70] De Ridder D., Elgoyhen A.B., Romo R., Langguth B. (2011). Phantom percepts: Tinnitus and pain as persisting aversive memory networks. Proc. Natl. Acad. Sci. USA.

[71] Husain F.T. (2016). Neural networks of tinnitus in humans: Elucidating severity and habituation. Hear. Res..

[72] Patil J.D., Alrashid M.A., Eltabbakh A., Fredericks S. (2023). The association between stress, emotional states, and tinnitus: A mini-review. Front. Aging Neurosci..

[73] Szczepek A.J., Mazurek B., Searchfield G.D., Zhang J. (2021). Neurobiology of Stress-Induced Tinnitus. The Behavioral Neuroscience of Tinnitus. Current Topics in Behavioral Neurosciences.

[74] Husain F.T., Khan R.A. (2023). Review and Perspective on Brain Bases of Tinnitus. J. Assoc. Res. Otolaryngol..

[75] Lanting C.P., De Kleine E., Van Dijk P. (2009). Neural activity underlying tinnitus generation: Results from PET and fMRI. Hear. Res..

[76] Cartocci G., Inguscio B.M.S., Giliberto G., Vozzi A., Giorgi A., Greco A., Babiloni F., Attanasio G. (2023). Listening Effort in Tinnitus: A Pilot Study Employing a Light EEG Headset and Skin Conductance Assessment during the Listening to a Continuous Speech Stimulus under Different SNR Conditions. Brain Sci..

[77] Newman C.W., Jacobson G.P., Spitzer J.B. (1996). Development of the Tinnitus Handicap Inventory. Arch. Otolaryngol. Head Neck Surg..

[78] Monzani D., Genovese E., Marrara A., Gherpelli C., Pingani L., Forghieri M., Rigatelli M., Guadagnin T., Arslan E. (2008). Validity of the Italian adaptation of the Tinnitus Handicap Inventory; focus on quality of life and psychological distress in tinnitus-sufferers. Acta Otorhinolaryngol. Ital..

[79] Moschen R., Fioretti A., Eibenstein A., Natalini E., Cuda D., Chiarella G., Rumpold G., Riedl D. (2018). Validation of the Italian Tinnitus Questionnaire Short Form (TQ 12-I) as a Brief Test for the Assessment of Tinnitus-Related Distress: Results of a Cross-Sectional Multicenter-Study. Front. Psychol..

[80] Khalfa S., Dubal S., Veuillet E., Perez-Diaz F., Jouvent R., Collet L. (2002). Psychometric Normalization of a Hyperacusis Questionnaire. ORL.

[81] Vernon J.A. (1987). Pathophysiology of tinnitus: A special case—Hyperacusis and a proposed treatment. Am. J. Otol..

[82] Jüris L., Andersson G., Larsen H.C., Ekselius L. (2013). Psychiatric comorbidity and personality traits in patients with hyperacusis. Int. J. Audiol..

[83] Blaesing L., Kroener-Herwig B. (2012). Self-reported and behavioral sound avoidance in tinnitus and hyperacusis subjects, and association with anxiety ratings. Int. J. Audiol..

[84] Sendesen E., Kılıç S., Erbil N., Aydın Ö., Turkyilmaz D. (2023). An Exploratory Study of the Effect of Tinnitus on Listening Effort Using EEG and Pupillometry. Otolaryngol. Head Neck Surg..

[85] Hallam R.S., Jakes S.C., Hinchcliffe R. (1988). Cognitive variables in tinnitus annoyance. Br. J. Clin. Psychol..

[86] Fioretti A., Tortorella F., Masedu F., Valenti M., Fusetti M., Pavaci S. (2015). Validity of the Italian version of Khalfa’s questionnaire on hyperacusis. Acta Otorhinolaryngol. Ital..

[87] Prunas A., Sarno I., Preti E., Madeddu F., Perugini M. (2012). Psychometric properties of the Italian version of the SCL-90-R: A study on a large community sample. Eur. Psychiatry.

[88] Derogatis L.R. (1994). Symptom Checklist-90-R: Administration, Scoring and Procedures Manual.

[89] Bersani F.S., Salviati M., Terlizzi S., Melcore C., Panico R., Romano G.F., Valeriani G., Altissimi G., Mazzei F., Testugini V. (2014). Tinnitus: Clinical experience of the psychosomatic connection. Neuropsychiatr. Dis. Treat..

[90] Spielberger C.D. (1984). State-Trait Anxiety Inventory: A Comprehensive Bibliography.

[91] Pedrabissi L., Santinello M. (1989). Verifica Della Validità Dello STAI Forma Y di Spielberger.

[92] Gasparre D., Pepe I., Laera D., Abbatantuono C., De Caro M.F., Taurino A., D’erasmo D., Fanizzi P., Antonucci L.A., Pantaleo A. (2023). Cognitive Functioning and Psychosomatic Syndromes in a Subjective Tinnitus Sample. Front. Psychol..

[93] Inguscio B.M.S., Nicastri M., Giallini I., Greco A., Babiloni F., Cartocci G., Mancini P. (2022). School wellbeing and psychological characteristics of online learning in families of children with and without hearing loss during the COVID-19 pandemic. Psychol. Sch..

[94] Yang S., Zhang M., Xu J., Wang L., Li Z., Zou F., Wu X., Wang Y. (2020). The electrophysiology correlation of the cognitive bias in anxiety under uncertainty. Sci. Rep..

[95] Eysenck M.W. (2000). A cognitive approach to trait anxiety. Eur. J. Pers..

[96] Soeter M., Kindt M. (2013). High trait anxiety: A challenge for disrupting fear memory reconsolidation. PLoS ONE.

[97] Guido G., Peluso A.M., Capestro M., Miglietta M. (2015). An Italian Version of the 10-Item Big Five Inventory: An Application to Hedonic and Utilitarian Shopping Values. Personal. Individ. Differ..

[98] Rammstedt B., John O.P. (2007). Measuring Personality in One Minute or Less: A 10-Item Short Version of the Big Five Inventory in English and German. J. Res. Pers..

[99] Oostenveld R., Praamstra P. (2001). The five percent electrode system for high-resolution EEG and ERP measurements. Clin. Neurophysiol..

[100] Di Flumeri G., Aricò P., Borghini G., Colosimo A., Babiloni F. A new regression-based method for the eye blinks artifacts correction in the EEG signal, without using any EOG channel. Proceedings of the 2016 38th Annual International Conference of the IEEE Engineering in Medicine and Biology Society (EMBC).

[101] Brunner C., Delorme A., Makeig S. (2013). Eeglab—An Open Source Matlab Toolbox for Electrophysiological Research. Biomed. Eng..

[102] Hubbard J., Kikumoto A., Mayr U. (2019). EEG Decoding Reveals the Strength and Temporal Dynamics of Goal-Relevant Representations. Sci. Rep..

[103] Klimesch W. (1999). EEG alpha and theta oscillations reflect cognitive and memory performance: A review and analysis. Brain Res. Rev..

[104] Skrandies W. (1990). Global field power and topographic similarity. Brain Topogr..

[105] Di Flumeri G., Giorgi A., Germano D., Ronca V., Vozzi A., Borghini G., Tamborra L., Simonetti I., Capotorto R., Ferrara S. (2023). A Neuroergonomic Approach Fostered by Wearable EEG for the Multimodal Assessment of Drivers Trainees. Sensors.

[106] Russo A.G., De Martino M., Mancuso A., Iaconetta G., Manara R., Elia A., Laudanna A., Di Salle F., Esposito F. (2020). Semantics-weighted lexical surprisal modeling of naturalistic functional MRI time-series during spoken narrative listening. Neuroimage.

[107] Inguscio B.M.S., Cartocci G., Sciaraffa N., Nasta C., Giorgi A., Nicastri M., Giallini I., Greco A., Babiloni F., Mancini P. (2021). Neurophysiological Verbal Working Memory Patterns in Children: Searching for a Benchmark of Modality Differences in Audio/Video Stimuli Processing. Comput. Intell. Neurosci..

[108] Turrini M., Cutugno F., Maturi P., Prosser S., Leoni F.A., Arslan E. (1993). Bisyllabic words for speech audiometry: A new italian material. Acta Otorhinolaryngol. Ital..

[109] Cartocci G., Scorpecci A., Borghini G., Maglione A.G., Inguscio B.M.S., Giannantonio S., Giorgi A., Malerba P., Rossi D., Modica E. (2019). EEG rhythms lateralization patterns in children with unilateral hearing loss are different from the patterns of normal hearing controls during speech-in-noise listening. Hear. Res..

[110] Attanasio G., Cartocci G., Covelli E., Ambrosetti E., Martinelli V., Zaccone M., Ponzanetti A., Gueli N., Filipo R., Cacciafesta M. (2012). The Mozart effect in patients suffering from tinnitus. Acta Otolaryngol..

[111] Shapiro S.S., Wilk M. (1965). An analysis of variance test for normality (complete samp). Biometrika.

[112] Mann H.B., Whitney D.R. (1947). On a Test of Whether One of Two Random Variables is Stochastically Larger Than the Other. J. Stat. Comput. Simul..

[113] Acharya M.S., Armaan A., Antony A.S. A comparison of regression models for prediction of graduate admissions. Proceedings of the 2019 International Conference on Computational Intelligence in Data Science (ICCIDS).

[114] Hazra A., Gogtay N. (2016). Biostatistics Series Module 6: Correlation and Linear Regression. Indian J. Dermatol..

[115] Kleinstäuber M., Weise C., Andersson G., Probst T. (2018). Personality traits predict and moderate the outcome of Internet-based cognitive behavioural therapy for chronic tinnitus. Int. J. Audiol..

[116] Langguth B., Kleinjung T., Fischer B., Hajak G., Eichhammer P.S.P.G., Sand P.G. (2007). Tinnitus severity, depression, and the big five personality traits. Prog. Brain Res..

[117] Mucci S., Geocze L., Abranches D.C., Antúnez A.E.A., Penido N.D.O. (2014). Systematic review of evidence on the association between personality and tinnitus. Braz. J. Otorhinolaryngol..

[118] Strumila R., Lengvenytė A., Vainutienė V., Lesinskas E. (2017). The role of questioning environment, personality traits, depressive and anxiety symptoms in tinnitus severity perception. Psychiatr. Q..

[119] Kusmaryono I., Wijayanti D., Maharani H.R. (2022). Number of Response Options, Reliability, Validity, and Potential Bias in the Use of the Likert Scale Education and Social Science Research: A Literature Review. Int. J. Educ. Methodol..

[120] Hall J.A., Bernieri F.J. (2001). Interpersonal Sensitivity: Theory and Measurement.

[121] Zhang Y., Sun Q. (2023). How Interpersonal Sensitivity Affects Depression under the COVID-19 Lockdown among College Students in South China: A Moderated Mediation Model. Psychol. Res. Behav. Manag..

[122] Bhatt J.M., Bhattacharyya N., Lin H.W. (2017). Relationships between tinnitus and the prevalence of anxiety and depression. Laryngoscope.

[123] Crocetti A., Forti S., Ambrosetti U., Bo L.D. (2009). Questionnaires to evaluate anxiety and depressive levels in tinnitus patients. Otolaryngol.—Head Neck Surg..

[124] Knowles K.A., Olatunji B.O. (2020). Specificity of trait anxiety in anxiety and depression: Meta-analysis of the State-Trait Anxiety Inventory. Clin. Psychol. Rev..

[125] Wang L.C., Chung K.K.H., Jhuo R.A. (2024). The relationships among working memory, state anxiety, and academic performance in Chinese undergraduates with SLD. Read. Writ..

[126] Nawaz D., Jahangir N., Khizar U., John H., Ilyas Z. (2021). Impact of anxiety on self-esteem, self-concept and academic achievement among adolescent. Elem. Educ. Online.

[127] El-Anzi F.O. (2005). Academic achievement and its relationship with anxiety, self-esteem, optimism, and pessimism in Kuwaiti students. Soc. Behav. Personal. Int. J..

[128] Pawlak-Osińska K., Kaźmierczak W., Kaźmierczak H., Wierzchowska M., Matuszewska I. (2013). Cortical activity in tinnitus patients and its modification by phonostimulation. Clinics.

[129] Moazami-Goudarzi M., Michels L., Weisz N., Jeanmonod D. (2010). Temporo-insular enhancement of EEG low and high frequencies in patients with chronic tinnitus. QEEG study of chronic tinnitus patients. BMC Neurosci..

[130] Vanneste S., van de Heyning M., De Ridder D. (2011). The neural network of phantom sound changes over time: A comparison between recent-onset and chronic tinnitus patients. Eur. J. Neurosci..

[131] Söderlund G., Sikström S., Smart A. (2007). Listen to the noise: Noise is beneficial for cognitive performance in ADHD. J. Child. Psychol. Psychiatry.

[132] Shulman A., Avitable M.J., Goldstein B. (2006). Quantitative electroencephalography power analysis in subjective idiopathic tinnitus patients: A clinical paradigm shift in the understanding of tinnitus, an electrophysiological correlate. Int. Tinnitus J..

[133] Shin S.H., Byun S.W., Lee Z.Y., Kim M.J., Kim E.H., Lee H.Y. (2022). Clinical Findings That Differentiate Co-Occurrence of Hyperacusis and Tinnitus from Tinnitus Alone. Yonsei Med. J..

[134] Kamiński J., Brzezicka A., Gola M., Wróbel A. (2012). Beta band oscillations engagement in human alertness process. Int. J. Psychophysiol..

[135] Kropotov J.D. (2010). Quantitative EEG, Event-Related Potentials and Neurotherapy.

[136] Vozzi A., Martinez Levy A., Ronca V., Giorgi A., Ferrara S., Mancini M., Capotorto R., Cherubino P., Trettel A., Babiloni F. (2023). Time-Dependent Analysis of Human Neurophysiological Activities during an Ecological Olfactory Experience. Brain Sci..

[137] Carter W.R., Johnson M.C., Borkovec T.D. (1986). Worry: An electrocortical analysis. Adv. Behav. Res. Ther..

[138] Field T., Ironson G., Scafidi F., Nawrocki T., Goncalves A., Burman I., Picken J., Fox N., Schanberg S., Kuhn C. (1996). Massage therapy reduces anxiety and enhances EEG pattern of alertness and math computations. Int. J. Neurosci..

[139] Jacobs G.D., Benson H., Friedman R. (1996). Topographic EEG mapping of the relaxation response. Biofeedback Self-Regul..

[140] Petruzzello S.J., Landers D.M. (1994). State anxiety reduction and exercise: Does hemispheric activation reflect such changes?. Med. Sci. Sports Exerc..

[141] Brunyé T.T., Patterson J.E., Wooten T., Hussey E.K. (2021). A critical review of cranial electrotherapy stimulation for neuromodulation in clinical and non-clinical samples. Front. Hum. Neurosci..

[142] Borghini G., Giorgi A., Ronca V., Mezzadri L., Capotorto R., Aricò P., Di Flumeri G., Babiloni F. Cooperation and mental states neurophysiological assessment for pilots’ training and expertise evaluation. Proceedings of the 2023 IEEE International Workshop on Technologies for Defense and Security (TechDefense).

[143] Sun J., Zhang X., Wang Y., Wang J., Li J., Cao F. (2020). The associations of interpersonal sensitivity with mental distress and trait aggression in early adulthood: A prospective cohort study. J. Affect. Disord..

[144] Langguth B., de Ridder D., Schlee W., Kleinjung T. (2024). Tinnitus: Clinical Insights in Its Pathophysiology—A Perspective. J. Assoc. Res. Otolaryngol..

[145] Landry E.C., Sandoval X.C.R., Simeone C.N., Tidball G., Lea J., Westerberg B.D. (2020). Systematic review and network meta-analysis of cognitive and/or behavioral therapies (CBT) for tinnitus. Otol. Neurotol..

[146] Barrenechea F.V. (2023). Efficacy of neurofeedback as a treatment for people with subjective tinnitus in reducing the symptom and related consequences: A systematic review from 2010 to 2020. Acta Otorrinolaringol..

